# Prevalence and risk factors of sarcopenia in community-dwelling older adults visiting regional medical institutions from the Kadoma Sarcopenia Study

**DOI:** 10.1038/s41598-020-76185-0

**Published:** 2020-11-05

**Authors:** Satoshi Kurose, Satoru Nishikawa, Takayasu Nagaoka, Masahiro Kusaka, Jun Kawamura, Yukari Nishioka, Shinji Sato, Hiromi Tsutsumi, Yutaka Kimura

**Affiliations:** 1 Department of Health Science, Kansai Medical University, 2-5-1 Shinmachi, Hirakata, Osaka, 573-1010 Japan; 2 Kadoma City Medical Association, Kadoma, Osaka, Japan; 3 Faculty of Human Science, Osaka International University, Moriguchi, Osaka, Japan; 4 Faculty of Health and Medical Science, Teikyo Heisei University, Tokyo, Japan

**Keywords:** Health care, Risk factors

## Abstract

This study aimed to investigate risk factors for sarcopenia in community-dwelling older adults visiting regional medical institutions. We retrospectively analyzed medical records of 552 participants (mean age: 74.6 ± 6.7 years, males 31.3%) who underwent body composition evaluation between March 2017 and December 2018 at one of 24 medical institutions belonging to the Kadoma City Medical Association in Japan. We collected the participant’s characteristics and laboratory data. Sarcopenia was diagnosed according to the Asian Working Group for Sarcopenia 2019. Sarcopenia, including severe sarcopenia, was detected in 22.3% of all participants, 17.3% of men, and 24.5% of women; rates increased with age. Multivariate logistic regression analysis revealed age (odds ratio [OR]: 2.12; 95% confidence interval [CI] 1.20–3.75), obesity (OR: 0.15; 95% CI 0.07–0.32), hypertension (OR: 0.44; 95% CI 0.25–0.76), certification of long term care (OR: 3.32; 95% CI 1.41–7.81), number of daily conversations (OR: 0.44; 95% CI 0.25–0.77), and malnutrition (OR: 2.42; 95% CI 1.04–5.60) as independent predictors of sarcopenia. Receiver operating characteristic curve analysis demonstrated that the cut-off for daily conversations defining sarcopenia was 4.8 persons. The prevalence of sarcopenia in this study was 22.3%. Besides traditional risk factors for sarcopenia, the number of daily conversations was an independent factor.

## Introduction

Sarcopenia is a syndrome characterized by progressive and generalized loss of skeletal muscle mass and strength that is associated with the risk of adverse outcomes such as physical disability, poor quality of life, and death^1,2^. Based on the definitions of sarcopenia provided in the European Working group on Sarcopenia in Older People (EWGSOP) and the International Working Group on Sarcopenia (IWGS) criteria, 1–29% of community-dwelling people aged ≥ 65 years and 14–33% of people living in care facilities meet the criteria for sarcopenia^3^. On the other hand, the prevalence of sarcopenia is estimated to be 6–12% in larger-scale studies^4–6^. However, the prevalence of sarcopenia depends on the definition applied and the attributes of the target population.

The Asian Working Group for Sarcopenia (AWGS) was established in 2014, and an algorithm for Asians was proposed^7^. In a previous study, the risk factors for sarcopenia were ageing and low weight, and it was also reported that the prevalence of sarcopenia increases due to lifestyle-related diseases such as metabolic syndrome and diabetes, wasting diseases such as chronic obstructive pulmonary disease, and malignant tumors^8–13^. However, since the relative influence of each of the risk factors for sarcopenia is unknown, a useful strategy for predicting its onset has not been established. Furthermore, sarcopenia studies of community-dwelling older adults often include highly motivated and interested groups, and this may not reflect real older adults. In addition, the AWGS was revised to the AWGS 2019^14^; the prevalence of sarcopenia under the new diagnostic criteria is unknown. In the future, medical and care costs are expected to increase further in advanced countries with an ageing population. Establishing early prevention methods for sarcopenia will enable quality interventions in each region.

Kadoma City, Osaka Prefecture, is located at the eastern end of Osaka City and is one of the satellite cities that form the commuter towns. Older adults comprise 28.9% of the population in Kadoma City, which exceeds that of Japan and Osaka Prefecture, and the rate is rapidly increasing^15^. There are many older adults living alone in Kadoma City, and ageing policies, including medical care and nursing care, are priorities in the region.

Community-dwelling older adults have access to regional medical institutions for medical examination and treatment, including treatment for sarcopenia. However, there are no multicenter studies on sarcopenia from regional medical institutions, and the relationship between lifestyle and sarcopenia is unclear. Therefore, the aim of this study was to investigate the characteristics and risk factors for sarcopenia among community-dwelling adults in cooperation with multicenter regional institutions.

## Methods

### Study design

This was a retrospective, cross-sectional, multicenter study on older community-dwelling adults who visited regional medical institutions.

### Subjects

**Figure 1:**
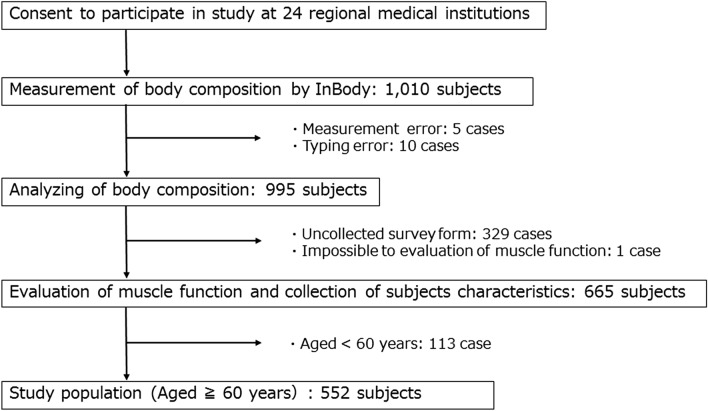
Flow chart of subject inclusion and exclusion.

The subjects were adults aged over 60 years old who visited each of the 24 medical institutions belonging to the Kadoma City Medical Association. We included 1010 subjects who underwent body composition evaluation between March 2017 and December 2018. The exclusion criteria included having a cardiac pacemaker, a neurological disease leading to an inability to walk independently, and cognitive or mental illness that was judged to make participation in this study difficult. We analyzed 552 participants with medical records that included muscle function measurements and physical and demographic characteristics (Fig. 1 and Table 1). This study was conducted in accordance with the Declaration of Helsinki, and all procedures were approved by the Ethical Committee of the Kansai Medical University (approval no. 1652). Written informed consent was obtained from all participants prior to the start of the study.

**Table 1:** Characteristics of participants.

	All n = 552	Men n = 173	Women n = 379	p-value
Age (years)	75.0 (60.0–94.0)	75.0 (61.0–91.0)	75.0 (60.0–94.0)	0.646
Height (cm)	154.0 (135.0–183.5.0)	163.2 (148.0–183.5)	151.0 (135.0–168.5)	< 0.001*
Body weight (kg)	55.6 (31.5–104.5)	65.6 (35.3–104.5)	52.4 (31.5–83.9)	< 0.001*
BMI (kg/m^2^)	23.3 (15.2–38.4)	24.5 (15.6–38.4)	22.7 (15.2–35.9)	< 0.001*
Obesity, n (%)	195 (35.3)	77 (44.5)	118 (31.1)	0.002*
Hypertension, n (%)	322 (55.1)	115 (66.9)	207 (55.1)	0.009*
Dyslipidemia, n (%)	265 (48.4)	82 (47.7)	183 (48.7)	0.829
Diabetes mellitus, n (%)	112 (20.4)	46 (26.7)	66 (17.6)	0.013*
Alcoholic drinks, n (%)	164 (30.7)	95 (55.9)	69 (18.9)	< 0.001*
Current smoker, n (%)	45 (8.4)	29 (17.1)	16 (4.4)	< 0.001*
Exercise habits, n (%)	242 (45.2)	78 (45.9)	1643 (44.9)	0.837

Data are expressed as median (min–max) or number (percent).

BMI, body mass index.

*Statistically significant.

### General evaluation

Characteristics of the participants were collected from the medical records of each medical institution. Obesity was defined as a body mass index (BMI) > 25 kg/m^2^; hypertension as systolic blood pressure ≥ 140 mmHg or diastolic blood pressure ≥ 90 mmHg; dyslipidemia as low-density lipoprotein (LDL) cholesterol ≥ 140 mg/dl, high-density lipoprotein (HDL) cholesterol < 40 mg/dl, or triglycerides ≥ 150 mg/dl; and diabetes as fasting plasma glucose ≥ 126 mg/dl, casual plasma glucose ≥ 200 mg/dl or glycated hemoglobin (HbA1c) ≥ 6.5%. Medical history was also collected from the medical records. In addition, alcohol consumption, smoking status, exercise habits, living situation, long-term healthcare insurance, and number of daily conversations were determined using questionnaires. Alcohol consumption was defined as the one or more alcohol consumptions per year, regardless of the amount. An exercise habit was defined as exercising at least once a week, regardless of duration. An exercise habit was defined as exercising at least once a week, regardless of duration. The number of daily conversations was investigated and the average number of conversations per day was assessed by the recall method.

### Laboratory examinations

Blood pressure was measured from the right arm of the seated subject after at least 15 min of rest. Non-fasting blood samples were collected to determine the serum levels of white blood cells, hemoglobin, plasma glucose, HbA1c, total cholesterol, HDL cholesterol, LDL cholesterol, triglycerides, aspartate aminotransferase (AST), alanine aminotransferase (ALT), γ-glutamyl transpeptidase (γ-GTP), blood urea nitrogen (BUN), creatinine, and albumin.

### Body composition, muscle strength, and performance

Body composition was measured using bioelectrical impedance analysis (InBody 470; InBody Japan, Tokyo, Japan). This system uses electrical currents of different frequencies to directly measure the amount of extracellular and intracellular water in the body. The body composition measurements included body weight, BMI, body fat mass, skeletal muscle mass, and skeletal muscle index (SMI). The SMI was defined as appendicular skeletal muscle divided by height squared in meters^16,17^.

Muscle strength and performance were measured using grip strength and gait speed, respectively. Grip strength was measured using a handgrip dynamometer (T.K.K.5401; Takei Scientific Instruments, Niigata, Japan). The measurements were performed twice for each hand, and the average of the highest value of each hand was recorded for analyses^18,19^. Gait speed was measured with the participant walking at a comfortable speed on a 6-m straight walkway, including 1-m for acceleration and deceleration. This speed was calculated as 4 m divided by the time to walk this distance in seconds (m/s).

According to the AWGS 2019 guidelines, SMI values of < 7.0 kg/m^2^ for men and < 5.7 kg/m^2^ for women were indicative of low muscle mass^14^. The cut-off point for grip strength was < 28 kg in men and < 18 kg in women, and the cut-off point for gait speed was < 1.0 m/s.

### Diagnosis of sarcopenia

We defined sarcopenia using the AWGS 2019 diagnostic algorithm. We classified low SMI + low muscle strength and low physical performance as severe sarcopenia, low SMI + low muscle strength or low physical performance as sarcopenia, and low muscle strength or low physical performance as possible sarcopenia^14^. The participants were divided into two groups (with or without sarcopenia) to compare the risk factors for sarcopenia. The sarcopenia group in this study included both severe sarcopenia and sarcopenia.

### Statistical analysis

Continuous and ordinal data are expressed as the median (min–max), and categorical data are expressed as incidences and percentages. The prevalence of sarcopenia is presented according to five age groups and sex. The Shapiro–Wilk test was used to identify the normality of data. The differences were analyzed using the Mann–Whitney U-test or the Chi-square test. A multivariate logistic regression analysis by likelihood ratio was carried out to determine the variables that were independently associated with the presence of sarcopenia. The dependent variable was sarcopenia, and the independent variables were the factors with a significant difference between groups and the adjustment factors. The continuous variables (as the independent variables in the analysis) were transformed to categorical ones by using their median values as a reference. Malnutrition was defined as total cholesterol < 150 mg/dl and/or albumin < 3.5 g/dl, and was analyzed as an adjustment factor. The cut-off value for sarcopenia was evaluated using the receiver operating characteristic (ROC) curve method. All statistical analyses were performed using SPSS 23.0J for Windows (IBM Corp., Armonk, NY, USA). P-values < 0.05 were considered statistically significant.

## Results

### Prevalence of sarcopenia

**Figure 2:**
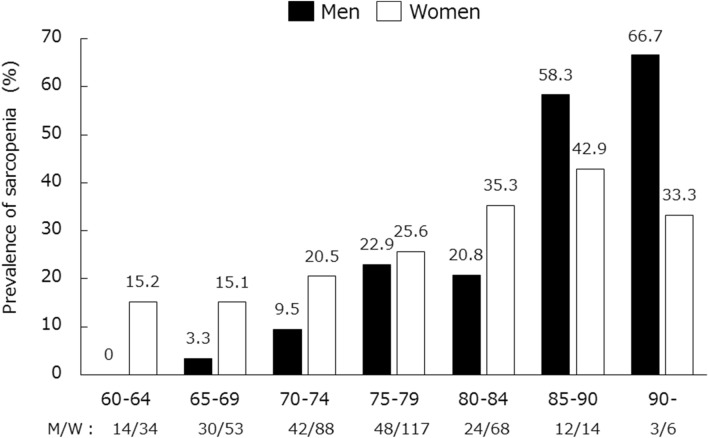
The prevalence of sarcopenia according to gender and age.

**Figure 3:**
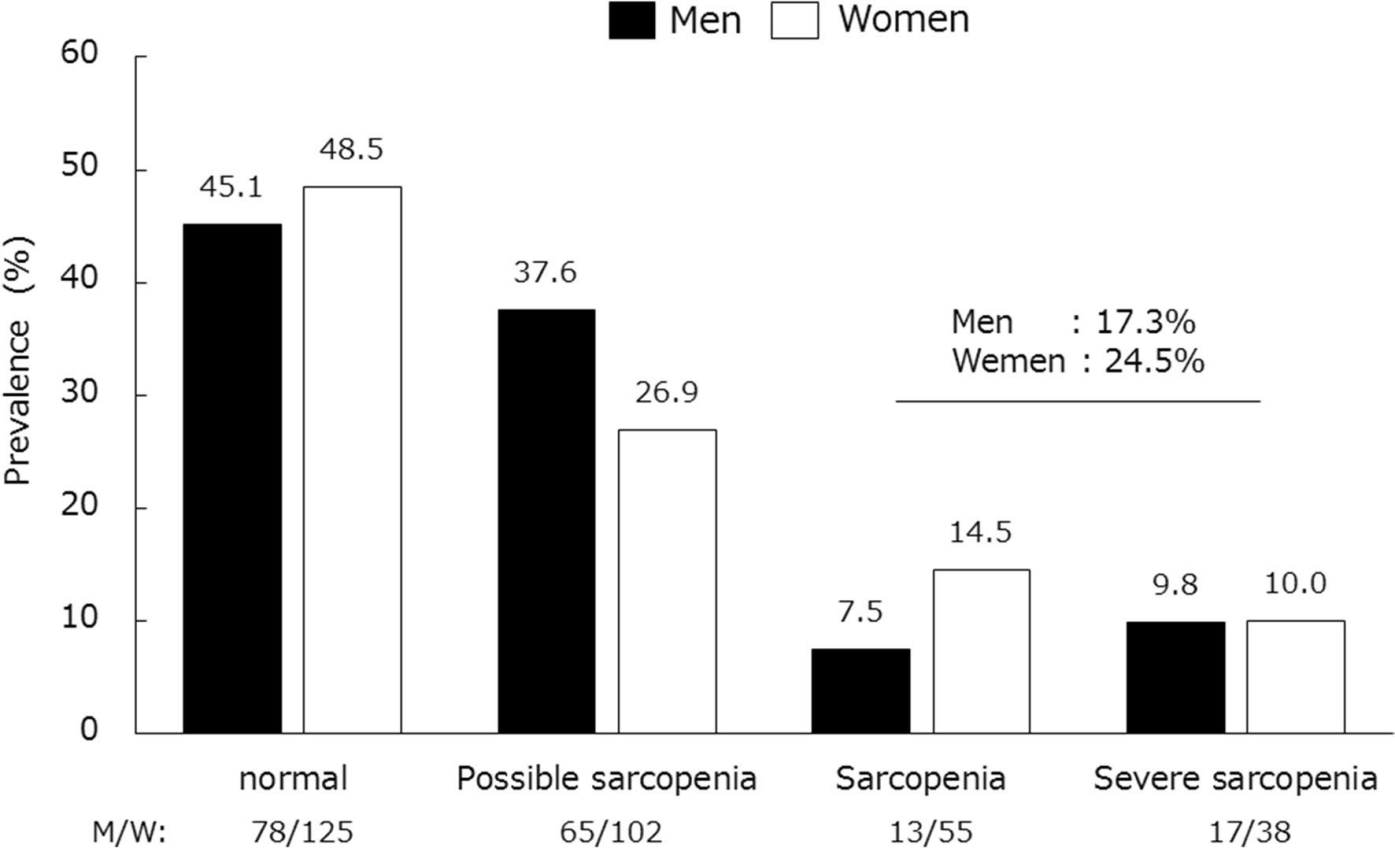
The prevalence of severe sarcopenia, sarcopenia and possible sarcopenia according to gender.

The prevalence of sarcopenia increased with age, especially in men over 85 years (Fig. 2). The prevalence of sarcopenia was 22.3% in all participants, 17.3% in men, and 24.5% in women (Fig. 3). Moreover, the prevalence of severe sarcopenia, sarcopenia, and possible sarcopenia in the overall cohort was 10.0%, 12.3%, and 30.3%, respectively.

### Risk factors for sarcopenia

Characteristics of the patients are given in Table 1. The sarcopenia group had significantly lower BMI; lower levels of hemoglobin, ALT, γ-GTP, creatinine, and albumin; lower rates of obesity, hypertension, bicycle use; and lower number of daily conversations than the non-sarcopenia group did (Table 2). Moreover, the sarcopenia group was significantly older; had higher total cholesterol, HDL cholesterol, and LDL cholesterol; and higher rates of osteoporosis, dementia, and certification of long-term care than the non-sarcopenia group did. Men with sarcopenia were significantly older; had significantly lower BMI, hemoglobin, triglycerides, and ALT levels; and exhibited lower levels of obesity, and higher rates of malignant tumors and certification of long-term care. Women with sarcopenia had significantly lower BMI and ALT, lower levels of obesity, hypertension, and lower number of daily conversations, and higher rates of osteoporosis and dementia.

**Table 2:** Comparison of patients with or without sarcopenia by gender.

	All n = 552			Men n = 173			Women n = 379		
	Sarcopenia	Non-Sarcopenia	p value	Sarcopenia	Non-Sarcopenia	p value	Sarcopenia	Non-Sarcopenia	p value
Number	123	429		30	143		93	286	
Age (years)	77.0 (61.0–94.0)	74.0 (60.0–93.0)	< 0.001*	79.0 (68.0–91.0)	73.0 (61.0–90.0)	< 0.001*	77.0 (61.0–94.0)	74.0 (60.0–93.0)	< 0.001*
Height (cm)	150.0 (138.0–170.0)	155.0 (135.0–183.5)	< 0.001*	161.0 (148.0–170.0)	165.0 (150.0–183.5)	< 0.001*	148.0 (138.0–164.0)	152.0 (135.0–168.5)	< 0.001*
Body weight (kg)	48.4 (31.5–73.8)	57.9 (35.2–104.5)	< 0.001*	57.1 (35.3–73.8)	67.2 (48.1–104.5)	< 0.001*	46.6 (31.5–60.0)	54.4 (35.2–83.9)	< 0.001*
BMI (kg/m^2^)	21.4 (15.2–32.8)	24.3 (16.1–38.4)	< 0.001*	21.4 (15.6–32.8)	25.0 (18.5–38.4)	< 0.001*	21.4 (15.2–28.7)	23.4 (16.1–35.9)	< 0.001*
Body fat mass (kg)	15.0 (4.8–36.4)	18.3 (5.4–44.5)	< 0.001*	13.9 (5.1–36.4)	19.1 (5.6–44.5)	0.001*	15.1 (4.8–27.5)	17.6 (5.4–39.9)	< 0.001*
Skeletal muscle mass (kg)	17.0 (13.0–25.3)	20.6 (14.6–38.0)	< 0.001*	22.6 (15.6–25.3)	26.6 (21.2–38.0)	< 0.001*	16.5 (13.0–20.7)	19.4 (14.6–29.5)	< 0.001*
SMI (kg/m^2^)	5.5 (3.2–7.0)	6.5 (4.7–10.1)	< 0.001*	6.6 (4.8–7.0)	7.6 (5.7–10.1)	< 0.001*	5.4 (3.2–5.7)	6.1 (4.7–8.7)	< 0.001*
Systolic blood pressure (mmHg)	130.0 (90.0–180.0)	130.0 (91.0–173.0)	0.858	134.0 (108.0–180.0)	130.0 (92.0–168.0)	0.109	130.0 (90.0–172.0)	131.0 (91.0–173.0)	0.225
Diastolic blood pressure (mmHg)	70.0 (44.0–125.0)	73.0 (40.0–114.0)	0.125	75.0 (50.0–91.0)	74.0 (50.0–114.0)	0.805	69.5 (44.0–125.0)	73.0 (40.0–103.0)	0.066
**Muscle function**									
Grip strength (kg)	17.0 (4.5–33.0)	22.5 (3.0–51.8)	< 0.001*	24.2 (5.5–33.0)	33.0 (14.0–51.8)	< 0.001*	16.0 (4.5–24.5)	20.5 (3.0–31.5)	< 0.001*
Gait speed (m/s)	0.90 (0.35–1.52)	1.13 (0.34–1.99)	< 0.001*	0.83 (0.35–1.19)	1.06 (0.43–1.78)	< 0.001*	0.92 (039–1.52)	1.14 (0.34–1.99)	< 0.001*
**Medical history**									
Obesity, n (%)	13 (10.6)	182 (42.4)	< 0.001*	5 (16.7)	72 (50.3)	0.001*	8 (8.6)	110 (38.5)	< 0.001*
Hypertension, n (%)	58 (47.5)	264 (62.0)	0.004*	16 (53.3)	99 (69.7)	0.083	42 (45.7)	165 (58.1)	0.037*
Dyslipidemia, n (%)	58 (47.5)	207 (48.6)	0.838	10 (33.3)	72 (50.7)	0.083	48 (52.2)	135 (47.5)	0.439
Diabetes mellitus, n (%)	26 (21.3)	86 (20.2)	0.786	11 (36.7)	35 (24.6)	0.177	15 (16.3)	51 (18.0)	0.717
Osteoarthritis of the knee, n (%)	28 (23.0)	74 (17.4)	0.163	5 (16.7)	17 (12.0)	0.484	23 (25.0)	57 (20.1)	0.315
Osteoarthritis of the hip, n (%)	3 (2.5)	11 (2.6)	0.939	1 (3.3)	3 (2.1)	0.687	2 (2.2)	8 (2.8)	0.739
Osteoporosis, n (%)	49 (40.2)	109 (25.6)	0.002*	5 (16.7)	11 (7.7)	0.126	44 (47.8)	98 (34.5)	0.022*
Dementia, n (%)	4 (3.3)	2 (0.5)	0.009*	1 (3.3)	1 (0.7)	0.222	3 (3.3)	1 (0.4)	0.018*
Malignant tumor, n (%)	5 (4.1)	11 (2.6)	0.380	4 (13.3)	5 (3.5)	0.028*	1 (1.1)	6 (2.1)	0.527
Fracture history, n (%)	47 (38.8)	136 (32.9)	0.228	10 (34.5)	55 (39.3)	0.628	37 (40.2)	81 (29.7)	0.061
Surgery history, n (%)	25 (20.5)	66 (15.5)	0.191	8 (26.7)	28 (19.7)	0.395	17 (18.5)	38 (13.4)	0.229
Low back pain, n (%)	56 (46.3)	183 (44.2)	0.461	14 (48.3)	57 (40.4)	0.435	42 (45.7)	126 (46.2)	0.933
Knee pain, n (%)	40 (33.1)	152 (36.7)	0.684	10 (34.5)	43 (30.5)	0.673	30 (32.6)	109 (39.9)	0.211
Users of hypnotics, n (%)	33 (27.3)	85 (20.5)	0.116	9 (31.0)	25 (17.7)	0.103	24 (26.1)	60 (22.0)	0.418
Certification of long-term care, n (%)	16 (13.3)	26 (6.3)	0.011*	5 (17.2)	5 (3.5)	0.004*	11 (12.1)	21 (7.7)	0.200
**Lifestyle**									
Alcoholic drinks, n (%)	33 (27.3)	131 (31.6)	0.359	18 (62.1)	77 (54.6)	0.461	15 (16.3)	54 (19.8)	0.461
Current smoker, n (%)	14 (11.6)	31 (7.5)	0.155	7 (24.1)	22 (15.6)	0.266	7 (7.6)	9 (3.3)	0.081
Exercise habits, n (%)	55 (45.5)	187 (45.2)	0.956	12 (41.4)	66 (46.8)	0.593	43 (46.7)	121 (44.3)	0.687
Living alone, n (%)	43 (35.0)	147 (34.3)	0.886	9 (30.0)	47 (32.9)	0.760	34 (36.6)	100 (35.0)	0.780
Users of bicycle, n (%)	64 (52.9)	267 (64.5)	0.021*	14 (48.3)	89 (63.1)	0.136	50 (54.3)	178 (65.2)	0.063
Daily conversation (person/ day)	3.0 (0.0–40.0)	4.0 (0.0–100.0)	0.020*	2.5 (0.0–40.0)	3.0 (0.0–100.0)	0.235	3.3 (0.0–25.0)	5.0 (0.0–50.0)	0.015*
**Laboratory examinations**									
WBC (/μl)	5500.0 (2900.0–10,010.0)	5450.0 (1900.0–11,930.0)	0.540	5960.0 (3190.0–10,010.0)	5800.0 (1900.0–9700.0)	0.910	5310.0 (2900.0–9120.0)	5300.0 2700.0–11,930.0)	0.773
Hemoglobin (g/dl)	12.8 (9.6–16.5)	13.2 (9.3–18.8)	0.005*	13.1 (10.6–16.5)	14.3 (9.4–18.8)	0.001*	12.6 (9.6–15.0)	12.8 (9.3–15.2)	0.790
Plasma glucose (mg/dl)	99.0 (70.0–339.0)	98.0 (65.0–366.0)	0.893	103.0 (83.0–224.0)	102.0 (65.0–366.0)	0.693	97.0 (70.0–339.0)	97.0 (70.0–297.0)	0.859
HbA1c (%)	5.7 (4.7–11.1)	5.7 (4.9–9.3)	0.319	6.0 (4.9–8.0)	5.7 (5.0–8.0)	0.189	5.7 (4.7–11.1)	5.7 (4.9–9.3)	0.665
Total cholesterol (mg/dl)	212.5 (135.0–317.0)	194.0 (123.0–303.0)	0.002*	199.0 (139.0–249.0)	187.0 (133.0–303.0)	0.471	219.0 (135.0–317.0)	199.5 (123.0–279.0)	0.002*
HDL cholesterol (mg/dl)	64.0 (37.0–101.0)	58.0 (21.0–190.0)	0.011*	54.0 (37.0–101.0)	50.0 (21.0–120.0)	0.053	64.5 (37.0–99.0)	62.0 (30.0–190.0)	0.237
LDL cholesterol (mg/dl)	115.0 (67.0–243.0)	108.0 (37.0–248.0)	0.003*	106.0 (73.0–163.0)	106.5 (37.0–219.0)	0.648	119.0 (67.0–243.0)	109.0 (50.0–248.0)	0.002*
Triglycerides (mg/dl)	103.0 (35.0–317.0)	114.0 (38.0–1244.0)	0.074	106.5 (49.0–175.0)	136.5 (38.0–1244.0)	0.024*	99.0 (35.0–317.0)	106.0 (38.0–589.0)	0.603
AST (IU/l)	22.0 (13.0–102.0)	22.0 (9.0–188.0)	0.391	23.0 (14.0–45.0)	23.5 (9.0–188.0)	0.764	22.0 (13.0–102.0)	22.0 (10.0–117.0)	0.446
ALT (IU/l)	15.5 (5.0–123.0)	17.0 (4.0–141.0)	0.001*	16.0 (6.0–40.0)	21.0 (4.0–132.0)	0.006*	15.0 (5.0–123.0)	16.0 (4.0–141.0)	0.043*
γ-GTP (IU/l)	19.0 (8.0–322.0)	23.0 (8.0–1742.0)	0.006*	25.5 (11.0–273.0)	28.0 (13.0–1742.0)	0.064	17.0 (8.0–322.0)	21.0 (8.0–320.0)	0.136
BUN (mg/dl)	17.2 (6.5–46.1)	16.6 (8.2–129.0)	0.742	17.2 (6.5–31.9)	17.3 (9.6–69.0)	0.613	17.2 (8.7–46.1)	16.0 (8.2–129.0)	0.337
Creatinine (mg/dl)	0.69 (0.39–7.66)	0.74 (0.40–8.17)	0.035*	0.84 (0.47–2.23)	0.91 (0.56–5.00)	0.160	0.67 (0.39–7.66)	0.67 (0.40–8.17)	0.499
Albumin (g/dl)	4.2 (1.1–4.7)	4.3 (1.3–4.9)	0.037*	4.2 (1.1–4.6)	4.3 (3.1–4.9)	0.103	4.2 (3.1–4.7)	4.2 (1.3–4.9)	0.174

Data are presented as median (min—max) or number (percent).

BMI, body mass index; SMI, skeletal muscle index; WBC, white blood cell; HDL, high-density lipoprotein; LDL, low-density lipoprotein; AST, aspartate aminotransferase; ALT, alanine aminotransferase; GTP, glutamyl transpeptidase; BUN, blood urea nitrogen.

*Statistically significant.

### Independent factors influencing sarcopenia

We performed multivariate logistic regression analysis using the likelihood ratio method and used a history of obesity to exclude multicollinearity of variables such as height, body weight, and BMI (Table 3). In the total study population, this analysis revealed that age (≥ 75 years) (odds ratio [OR]: 2.12; 95% confidence interval [CI] 1.20–3.75), obesity (OR: 0.15; 95% CI 0.07–0.32), hypertension (OR: 0.44; 95% CI 0.25–0.76), certification of long-term care (OR: 3.32; 95% CI 1.41–7.81), the number of daily conversations (with < 5 persons) (OR: 0.44; 95% CI 0.25–0.77) and malnutrition (total cholesterol < 150 mg/dl and/or albumin < 3.5 g/dl) (OR: 2.42; 95% CI 1.04–5.60) were all independent predictors of sarcopenia. The independent predictors for male sarcopenia were age (OR: 6.04; 95% CI 1.98–18.48), Obesity (OR: 0.23; 95% CI 0.07–0.77) and anemia (hemoglobin < 13.0 mg/dl) (OR: 3.44; 95% CI 1.15–10.26) after adjustment for malnutrition. The independent predictors for female sarcopenia were obesity (OR: 0.18; 95% CI 0.07–0.43) and the number of daily conversations (OR: 0.51; 95% CI 0.28–0.96) after adjustment for malnutrition.

**Table 3:** Multivariate logistic regression analysis of independent predictors of sarcopenia.

	Adjusted odds ratio	95% confidence interval	p value
**A. All**^**a,b**^			
Age	2.12	1.20–3.75	0.010*
Obesity	0.15	0.07–0.32	< 0.001*
Hypertension	0.44	0.25–0.76	0.003*
Certification of long-term care	3.32	1.41–7.81	0.006*
Number of daily conversations	0.44	0.25–0.77	0.004*
Malnutrition	2.42	1.04–5.60	0.039*
**B. Men**^**c,d**^			
Age	6.04	1.98–18.48	0.002*
Obesity	0.23	0.07–0.77	0.017*
Anemia	3.44	1.15–10.26	0.027*
**C. Women**^**e,f**^			
Obesity	0.18	0.07–0.43	< 0.001*
Number of daily conversations	0.51	0.28–0.96	0.037*

^a^Independent variables: age (≥ 75 years), obesity, hypertension, osteoporosis, dementia, anemia (hemoglobin: male < 13.0 g/dl, female < 12.0 g/dl), certification of long-term care, use of bicycles, number of daily conversations (≥ 5 persons).

^b^Adjusted variables: gender, malnutrition (total cholesterol < 150 mg/dl and/or albumin < 3.5 g/dl).

^c^Independent variables: age (≥ 75 years), obesity, malignant tumor, anemia (hemoglobin < 13.0 g/dl), certification of long-term care.

^d^Adjusted variables: malnutrition (total cholesterol < 150 mg/dl and/or albumin < 3.5 g/dl).

^e^Independent variables: age (≥ 75 years), obesity, hypertension, osteoporosis, dementia, number of daily conversations (≥ 5 persons).

^f^Adjusted variables: malnutrition (total cholesterol < 150 mg/dl and/or albumin < 3.5 g/dl).

*Statistically significant.

**Figure 4:**
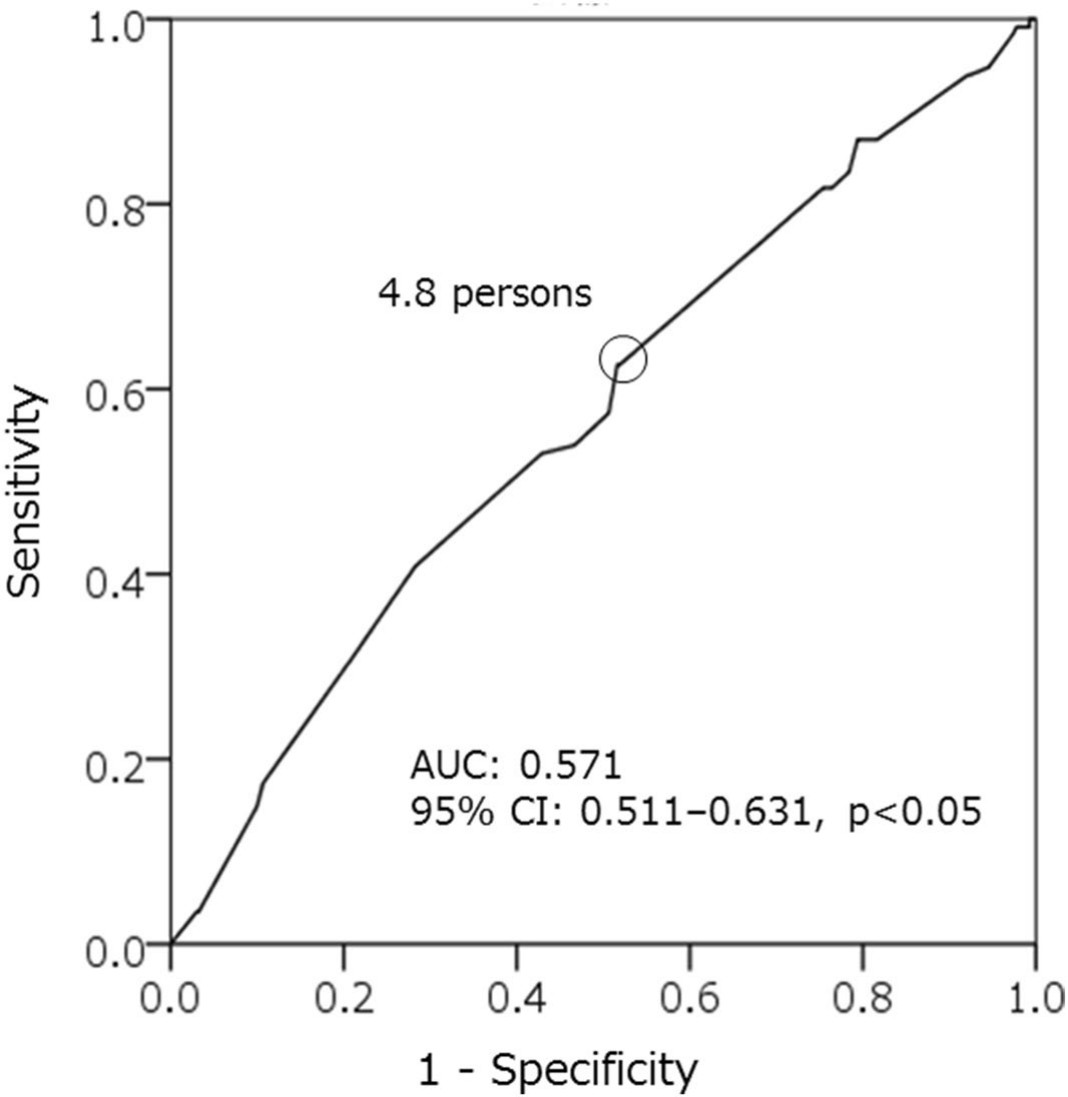
ROC curve demonstrating the diagnostic value of daily conversation. Receiver operating characteristic (ROC) curve analysis demonstrates the statistically significant influence of the number of daily conversations on sarcopenia (area under the curve was 0.571 with 95% CI of 0.511–0.631, P < 0.05).

The ROC curve analysis demonstrated that a lower number of daily conversations (with < 5 persons) was significantly correlated with sarcopenia (area under the curve was 0.571 with a 95% CI of 0.511–0.631, p < 0.05). The sensitivity and specificity for 4.8 persons for diagnosis of sarcopenia were 62.6% and 48.1%, respectively (Fig. 4).

## Discussion

This study was a multicenter cross-sectional study including 24 regional medical institutions. The overall prevalence of sarcopenia was 22.3%, being 17.3% in men and 24.5% in women. Rates of sarcopenia increased with age in men older than 60 years, and after 85 years of age, the prevalence of sarcopenia in men exceeded that in women. On the other hand, sarcopenia was observed in women from 60 years, indicating that early prevention of sarcopenia is important.

In a previous study, the prevalence of Japanese sarcopenia in individuals aged 65 to 89 years according to the EWGSOP algorithm for sarcopenia was reported to be 21.8% in men and 22.1% in women, and the prevalence of sarcopenia increased with age in both men and women older than 75 years^20^. We found that there were significant numbers of women aged 60–84 years with sarcopenia. The rates for both sexes with regard to age were similar to the results of the previous study.

The EWGSOP algorithm defines the grip strength cut-offs as 30 kg for men and 20 kg for women, which may be the reason for the higher prevalence compared with AWGS. It has been reported that the prevalence of sarcopenia in older adult Japanese individuals according to the AWGS algorithm was 9.6% in men and 9.2% in women, and there were differences depending on the diagnostic criteria and region^21^. Many of the previous studies include subjects who have participated consciously through local public relations and solicitation and have an interest in being tested for sarcopenia. Therefore, they may not reflect a representative sample of the older adult Japanese population. The strength of this present study was that it excluded participant selection bias because there are a wide range of older adults who attend regional medical institutions for medical examination and periodic health check-ups. In addition, this study used the AWGS 2019 and provided useful information reflecting the actual conditions in the region.

Because sarcopenia has a poor prognosis, the development of prevention methods is an important issue^22^. The prevention of sarcopenia can be implemented by detection of possible sarcopenia, which are the early stages of sarcopenia, so that early intervention for these patients can be implemented^23^. In the present study, the prevalence of possible sarcopenia was 30.3%. Moreover, 52.6% of individuals with severe sarcopenia, sarcopenia, and possible sarcopenia showed low muscle strength and/or low muscle performance. These results suggest the usefulness of periodic body composition, muscle strength, and performance measurements for early detection and intervention at earlier stages of sarcopenia may contribute to primary prevention. In addition, the rate of certification of long-term care in subjects classified with sarcopenia was 13.3%. In other words, 86.7% of the sarcopenia group were not certified for long-term care, indicating that they will need to access continuous intervention service other than medical and nursing care insurance.

Independent risk factors for overall sarcopenia were age (≥ 75 years), lower rates of obesity and hypertension, higher rates of certification of long-term care and malnutrition, and a low number of daily conversations (with < 5 persons). The independent factors for men were age, lower rates of obesity and higher rates of anemia, and the independent factors for women were lower rates of obesity and low number of daily conversations. Although sex was not identified as an overall independent factor, only lower rate of obesity was a risk factor common to both sexes. Therefore, sex differences should be considered when assessing patients for sarcopenia.

Interestingly, a novel finding of this study was that the number of daily conversations (with < 5 persons) is an independent factor for sarcopenia in addition to the traditional risk factors^8–13^. It has been reported that the number of daily conversations is a preventive factor for dementia^24^, and that social frailty was an independent factor for muscle weakness^25^. However, there are no results concerning the influence of the number of daily conversations in previous sarcopenia studies. In this study, the daily conversation was investigated by a proposal from a regional medical institution (Kadoma City Medical Association) that care for the living environment and social interaction of the patient^26^. As a result, the number of daily conversations was an independent factor for sarcopenia with a cut-off of 4.8 persons. When the number of daily conversations with different people was more than 5 persons, this indicated that the person is going out and seeking social interaction since this would be difficult to achieve by staying at home or talking only with one’s own family. The number of daily conversations is an index that comprehensively reflects physical, mental, and social function, and it may be an important marker for early detection of sarcopenia.

Physical activity and exercise are generally related to the maintenance and improvement of muscle strength and muscle mass^27,28^. Although use of a bicycle was not an independent factor in this study, there was a significant difference in bicycle use in the sarcopenia and non-sarcopenia groups in this study. In a previous study investigating the relationship between leisure sports and life expectancy, it was reported that group exercise and sports extended life expectancy to a greater degree than exercises that are performed alone, such as walking^29^.

Given the above findings, it is suggested that sarcopenia can be prevented by engaging in social activities to increase conversation opportunities in daily life, which may take the form of social events or exercise that involves a group of people.

There were several limitations to this study. First, it was a cross-sectional study, and therefore, the causal relationship between sarcopenia and the various influencing factors remains unknown. Therefore, a prospective intervention study that includes the risk factors found in this study is needed to confirm this relationship. Second, the participants responded without defining their exercise habits. An exercise habit, according to the National Health and Nutrition Examination Survey, is defined as participating in exercise for 30 min more than twice a week. A high proportion of study participants exhibited an exercise habit (41.2%). However, we did not define exercise habit; therefore, participants may have indicated that they have an exercise habit even if they exercised once a week or for a short period of time. Therefore, it is difficult to compare our results with other studies. Third, the result of the ROC analysis of the number of daily conversations is not sufficiently robust and the optimal cut off value remains controversial. However, daily conversations and physical activity may reportedly help prevent dementia in older Japanese adults^24^. Suggesting that a large number of daily conversations may have a positive effect on physical function at least. Finally, the evaluation of the number of daily conversations was an approximation of the average using the recall method. It should be noted that there is no gold standard to assess the number of daily conversations as it depends on the subjective responses of participants. In addition, the conversation partner and content were unknown. Although the number of daily conversations was evaluated in this study, the quality of the conversation, including who the conversation partner was, the content and the duration may affect sarcopenia. However, it has been reported that the use of communication robots for older adults improved their activity levels, motivation, and sociality^30,31^. Therefore, it is suggested that the act of conversation itself is beneficial.

In conclusion, the prevalence of sarcopenia among local residents who visited the medical institutions in this study was 22.3%. The most interesting finings of our study was that a low number of daily conversations (with < 5 persons) was an independent risk factor for sarcopenia in both men and women. We suggest that older adults be encouraged to engage in social activities to increase conversation opportunities in daily life, whether that is through social events or exercise activities that involve a group of people. This may prevent the progression of sarcopenia and lead to a higher quality of life for older adults.
